# Autism With and Without Regression: A Two-Year Prospective Longitudinal Study in Two Population-Derived Swedish Cohorts

**DOI:** 10.1007/s10803-018-03871-4

**Published:** 2019-02-04

**Authors:** Lucy Thompson, Christopher Gillberg, Sara Landberg, Anne-Katrin Kantzer, Carmela Miniscalco, Martina Barnevik Olsson, Mats A. Eriksson, Elisabeth Fernell

**Affiliations:** 1 Gillberg Neuropsychiatry Centre, Institute of Neuroscience and Physiology, University of Gothenburg, Gothenburg, Sweden; 2 Institute of Health and Wellbeing, University of Glasgow, Glasgow, Scotland, UK; 3 PRIMA Child and Adult Psychiatry, Stockholm, Sweden; 4 Department of Women’s and Children’s Health, Karolinska Institutet, Stockholm, Sweden

**Keywords:** Autism, ASD, Regressive autism, Non-regressive autism, Intellectual developmental disorder, Developmental language disorder

## Abstract

Two community-based cohorts of children with autism spectrum disorder, examined using similar assessment protocols, were pooled (n = 301) and subdivided according to history of regression. Those with regression (n = 62), 20.5% of the combined cohort, were contrasted with those without regression (n = 241) at first assessment (age range 19–60 months) and at 2-year follow-up on a range of measures. The regression group was significantly more functionally impaired, with regard to intellectual function (p < .001), language development (p < .001), and to severity of autism (p < .01) at both T1 and T2. Only 14 (23.3%) had a clearly identified underlying etiology [24 (18.6%) in the non-regressive group]. There were no significant differences between those who had regressed ‘from normal’ and those who had regressed ‘from low’ functioning.

## Introduction

Many children with autism spectrum disorder (ASD) (American Psychiatric Association 2013) have shown mild or marked developmental problems from early childhood (Coleman and Gillberg 2012). However, regression in a subgroup of individuals with autism has been recognized for decades, indeed for more than a century (Heller 1908), but very little population-based research on the phenomenon has been published. Where research purports to be population-based, there are usually still issues with case ascertainment as samples do not derive from whole population screening (i.e., rely on existing diagnoses within a population (e.g., Baird et al. 2008). The few systematic studies that have been published seem to indicate that regression in autism is (a) not uncommon, but the relative prevalence is not known, and (b) associated with high rated of intellectual developmental disorder (IDD) in follow-up studies (Bradley et al. 2016; Gadow et al. 2017; Shinnar et al. 2001; Stefanatos 2008). Some in-depth clinically based studies have suggested a temporal association between regression in autism and epilepsy and vice versa (Gadow et al. 2017; Tuchman et al. 2010), but this has not been confirmed in population-based surveys (Fernell et al. 2010; Kantzer et al. 2013). The role of possible neuroinflammation in “regressive autism” has been the subject of speculation (Pardo et al. 2013) and evidence of underlying autoimmune processes and neuroinflammation (Scott et al. 2014, 2017) has been suggested. However, very little, if any, systematic evidence is available (Kern et al. 2016).

It is not clear if regressive autism/autism with regression is the same or different than Heller’s disease or childhood disintegrative psychosis/disorder (Coleman and Gillberg 2012). The “typical” reported age of regression in cases described as regressive autism seems to be about 18–24(30) months of age (Tuchman 2003; Baird et al. 2008; Stefanatos 2008; Hoshino et al. 1987) whereas most cases (albeit certainly not all) of disintegrative disorder (which no longer appears in the diagnostic manuals) have been reported to show regression after age 30 months (e.g. (Russo et al. 1996; Charan 2012)). It is also not known to what extent relatively more well-defined disorders with regression—such as Rett syndrome, with a clear genetic cause, and Landau-Kleffner-syndrome, likely, in some cases, with a genetic cause (syndromes which are often diagnosed with autism)—overlap or indeed account for some cases of regressive autism.

In the present report we have combined two of the largest in-depth assessed community/population-based preschool autism cohorts with a view to establishing (a) the relative prevalence of regression in autism, (b) typical associated preschool age problems in regressive as compared with non-regressive autism, including other Early Symptomatic Syndromes Eliciting Neurodevelopmental Clinical Examination (ESSENCE) (Gillberg 2010) characteristics (e.g., hyperactivity), (c) possible predictors, mediators and moderators of regression in autism, including pre- and perinatal factors, and (d) specific identified underlying etiologies in the Stockholm group. Given the lack of previous systematic representative studies in the field, our study sets out to be descriptive rather than hypothesis-driven.

## Methods

Data from two community-based cohorts were used in the present study. The cohorts were recruited in Stockholm and in Gothenburg, the two biggest cities in Sweden.

### Stockholm Cohort

The Stockholm cohort comprised originally 208 children, although one parent has since withdrawn consent for their data to be used, so the sample reported here is 207 (32 girls, 175 boys). They were all cases diagnosed with ASD, according to the DSM-IV (American Psychiatric Association 1994), at their local Child and Adolescent Mental Health Service (CAMHS) or neuropediatric clinic from 2005 to 2008. They are considered to be representative of all children with ASD in Stockholm at the time of study, except those with very severe co-occurring complex neurological syndromes and profound intellectual disability (n = 27) and of those with parents not able to communicate in Swedish or English for the purpose of the study (n = 15). In addition, 37 families had declined participation in the study since their child had recently had a clinical comprehensive assessment at their local CAMHS (Fernell et al. 2010). The male to female gender ratio was 5.5:1. Age at diagnosis was between 2 and 4.5 years and they had all been referred to the specialized habilitation center in Stockholm, the Autism Center for Young Children (ACYC), to receive intervention. All children and parents met with a pediatrician/child and adolescent psychiatrist and were assessed in-depth at the ACYC at two time points; at Time 1 (T1) shortly after the child had received the diagnosis of ASD and at Time 2 (T2) after 2 years, during which the child had received intervention. At follow-up 197 children participated with their parents (Fernell et al. 2010, 2011).

### Gothenburg Cohort

The Gothenburg cohort comprised originally 129 children. Between 2009 and 2011, all 2.5 year-old children in Gothenburg, Sweden underwent a new autism-screening program at the Child Health Care centers (Nygren et al. 2012). Children who were ‘screen positive’ for suspected ASD were referred to Child Neuropsychiatry Clinic (CNC) (Nygren et al. 2012; Kantzer et al. 2013). The cohort is clearly population-representative as it is based on general population screening and detailed assessment and follow-up of all screen positive cases. All children were assessed by an experienced multi-disciplinary team consisting of a neuropsychiatrist, a neuropsychologist, a speech-language pathologist and a preschool educational diagnostician at study intake (T1). In all, 129 children were assessed for suspected ASD (at a mean age of 2.9 years); the DSM-IV ASD diagnosis was confirmed in 100 children and the remaining 29 presented with sub-threshold symptoms of autism, ADHD and developmental language disorder (DLD). At follow-up, 2 years later (T2), 96 of the original 129 children participated with their parents (Kantzer et al. 2018).

### Measures and Assessment Procedures in the Two Cohorts

At T1 and T2, a structured parental interview was used, including questions about hereditary factors, the child’s development; such as age when the child started unsupported walking, whether toe walking had existed, activity level, whether there had been feeding problems during the child’s first 2 years (gathered from CHC records in Stockholm) and if the child had previous or ongoing treatment for epilepsy.

There was also an evaluation of the child’s severity of ASD at T1 and T2: in Stockholm this was done using the Autistic Behavior Checklist (ABC) score (Krug et al. 1980); in Gothenburg the Autism Diagnostic Observation Schedule - Generic (ADOS-G) (Lord et al. 2000) was applied. Specific questions were asked regarding whether the child had a regressive type of autism, defined as loss of expressive language skills (loss of 5 or more words that had been used communicatively) in connection with the onset of autism. Although the same regression criteria were applied to both cohorts, the classification of cases was slightly different in the two cohorts: Gothenburg cases were classified according to the timing of regression (i.e., before or after 24 months) whereas Stockholm cases were classified according to functional level (i.e., from a “normal” or “low” level, mostly close to the same time, around the age of 24 months) (Fernell et al. 2010). In the Stockholm cohort, regression was defined as loss of more than five spoken words used communicatively in children more than 15 months of age. In children younger than 15 months, regression was determined when there was a clear indication of loss of social interest and contact. Parents were interviewed (in close temporal connection to the first diagnostic assessment) regarding a history of regression in the child and this information was compared to available data in CHC and medical records. Consistency was required between parental information and the notes in the records from CHC. In one group, parents had reported that their child’s general development had been “normal” before language regression and in one group parents had had concerns regarding their child’s development before regression.

Obstetric records were checked for maternal disorders during pregnancy and pregnancy complications and for perinatal events. Records from the child’s Health Care Center (CHC) were checked with regard to growth charts and developmental milestones.

A physical examination of the child was performed, including an evaluation of muscular tone, physical anomalies, head circumference and skin. Parents were asked about etiological diagnoses established and were offered that their child could have a genetic test (chromosomal microarray, see (Eriksson et al. 2015)) (Gothenburg results still in process, so only Stockholm will be reported here).

Children were assessed by a speech and language pathologist at T1 and T2 and a new cognitive test was performed by a psychologist at T2. One of the measures used to assess the child’s level of speech and language was the PARIS schedule (the Paris Autism Research In Sib-pairs; detailed in Fernell et al. 2010) that classified expressive language skills into one of five levels: no speech at all, a few single words, a few communicative sentences, and phrase speech with or without echolalia.

General cognitive/developmental level was assessed using either the Griffiths’ test (Alin-Åkerman and Nordberg 1980), the Wechsler Preschool and Primary Scale of Intelligence (WPPSI-III) (Wechsler 2005), or the Merrill–Palmer test (Roid and Sampers 2004). IDD was diagnosed when Intellectual Quotient (IQ)/Developmental Quotient (DQ) was below 70, combined with a corresponding low general adaptive function. borderline intellectual functioning (BIF) was diagnosed when the IQ/DQ was between 70 and 84. Average intellectual functioning (AIF) was diagnosed when IQ/DQ was 85 or above. The child’s adaptive function was assessed at T1 and T2 by a parental interview according to the Vineland Adaptive Behavior Scales-II (VABS) (Sparrow et al. 2005).

### Combined Sample

All data were scrutinized, completed (e.g., from medical records) and compared in 2017 to examine regressive cases in both cohorts. The two cohorts were combined for some analyses because they were very similar in terms of demographics, symptoms and proportion of regressive/non-regressive cases. The total sample consisted of 303 participants (49 girls, 254 boys). Tables 1, 2, 6 and 7 indicate the variables on which comparisons could validly be made.

**Table 1:** Gothenburg and Stockholm samples: background factors

Variables	Gbg (n = 96)^a^	Stk (n = 207)^b^	Statistic	*p*
Male (F (%))	79 (82.3)	175 (84.5)	0.245^c^	0.621
Age at T1-years (M (SD))	3.0 (0.53)	3.8 (0.68)	-11.165^d^	< **0.001**
Age at T2-years (M (SD))	5.2 (0.53)	5.5 (0.73)	-3.364^d^	**0.001**
Parents have different ethnicity (F (%))	22 (23.2)	44 (21.4)	0.123^c^	0.726
Any NDD in first degree relative? (F (%))	48 (50.0)	71 (34.29)	6.779^c^	**0.009**
Number siblings (M (SD))	1.62 (0.96)	1.14 (0.81)	4.40^d^	< **0.001**
Birth order (M (SD))	2.09 (0.96)	1.63 (0.77)	1.399^d^	< **0.001**
Any maternal disease in pregnancy? (F (%))^e^	30 (31.3)	87 (48.6)	7.698^c^	**0.006**
Gestational age (M (SD))	39.2 (2.50)	39.1 (2.16)	0.367^d^	0.714
Born at term (37–41 weeks)? (F (%))	69 (71.9)	178 (86.0)	8.735^c^	**0.013**
Caesarean section? (F (%))	17 (17.7)	59 (29.2)	4.529^c^	0.033
Neonatal care? (F (%))	20 (20.8)	39 (20.0)	0.028^c^	0.868
Age walking (M (SD))	13.8 (2.92)	14.5 (4.69)	− 1.575^d^	0.116
Toe walking? (F (%))	28 (29.2)	70 (33.7)	0.648^c^	0.421
Feeding problems first 2 years? (F (%))	46 (47.9)	38 (18.4)	28.599^c^	< **0.001**

Significant p values (p < .01) are highlighted in bold

NDD: Neurodevelopmental Disorder

^a^Specific N for each variable ranged from 88 to 96

^b^Specific N for each variable ranged from 179 to 207

^c^chi-squared test

^d^t-test

^e^Gbg data from single variable; Stk data compiled from more detailed data: chronic condition, acute condition, and/or pharmacological intervention in pregnancy

**Table 2:** Gothenburg and Stockholm samples: measurements and outcomes

Variables	Gbg (n = 96)^a^	Stk (n = 207)^b^	Statistic	*p*
Measurements				
Birthweight (M (SD))	3395.96 (675.78)	3518.24 (623.43)	− 1.520^d^	0.130
Birthweight SD for gestational age (F (%))			0.034^c^	0.983
≤ 2SDs	2 (2.2)	4 (2.0)		
Within ± 2SDs	87 (95.6)	194 (95.6)		
≥ 2SDs	2 (2.2)	5 (2.5)		
Head Circumference (HC) Birth (M (SD))	34.69 (2.59)	35.08 (1.87)	− 1.438^d^	0.152
HC Birth SD for gestational age (F (%))			2.016 ^c^	0.365
≤ 2SDs	0 (0)	3 (1.5)		
within ± 2SDs	87 (97.8)	189 (97.4)		
>+ 2SDs	2 (2.2)	2 (1.0)		
HC T1 SD (F (%))			4.620^c^	0.099
≤ 2SDs	1 (1.3)	2 (1.0)		
within ± 2SDs	71 (89.9)	196 (96.1)		
>+ 2SDs	7 (8.9)	6 (2.9)		
Assessment outcomes				
Language T1 − minimal or none (F (%))	49 (51)	94 (45.4)	0.834^c^	0.361
Language T2 − minimal or none (F (%))	12 (12.5)	56(28.4)	9.186^c^	**0.002**
Activity level T1 − hyper/hypo (F (%))	36/3 (37.5/3.1)	87/7 (42.0/3.4)	0.612 ^c^	0.736
Activity level T2 − hyper/hypo (F (%))	36/3 (37.5/3.1)	103/8 (52.3/4.1)	6.393 ^c^	0.041
Epilepsy T1 (F (%))	7 (7.3)	13 (6.3)	0.109^c^	0.741
Epilepsy T2 (F (%))	5 (5.2)	17 (8.5)	1.043^c^	0.307
Intellectual level T1 − BIF/IDD (F (%))	24/30 (25/31.3)	81/78 (39.3/37.9)	14.402^c^	**0.001**
Intellectual level T2 − BIF/IDD (F (%))	25/25 (26/26)	29/93 (14.9/47.7)	13.490^c^	**0.001**
Vineland total T1 (M (SD))	76.48 (9.61)	68.81 (10.72)	5.852^d^	< **0.001**
Vineland total T2 (M (SD))	75.27 (13.36)	69.99 (14.80)	2.891^d^	**0.004**
ASD dx T1-autism/autistic-like (F (%))	53/23 (55.2/24.0)	137/47 (67.5/23.2)	8.143^c^	**0.017**
ASD dx T2-autism/autistic-like (F (%))	49/30 (51.0/31.3)	104/72 (52.8/36.5)	3.031^c^	0.220

Significant p values (p < .01) are highlighted in bold

*BIF* borderline intellectual functioning, *IDD* intellectual development disorder, *ASD* autism spectrum disorder

^a^Specific N for each variable ranged from 79 to 96

^b^Specific N for each variable ranged from 194 to 207

^c^chi-squared test

^d^t-test

### Ethics

The Stockholm study was approved by the ethics committee at Karolinska University Hospital, Stockholm, reference number 2006/61–31/2. The Gothenburg study was approved by the Regional Ethics Review Board, University of Gothenburg, Sweden, registration number 494-08. Informed consent was obtained from at least one of the parents/responsible carers for each child in both cohorts.

## Results

### Description of and Comparison Across the Two City Cohorts

Although the two cohorts are very similar, there are some points on which they differ. With reference to Tables 1 and 2, children in the Gothenburg cohort were significantly younger (T1 3.0 years; T2 5.2 years) on average than those in the Stockholm cohort (T1 3.8 years; T2 5.5 years), most likely due to the different methods of ascertainment of each cohort (i.e., Gothenburg came from a routine whole-population screening program, carried out at the age of 2.5 years). Around a fifth of each cohort (23.2% and 21.4%, respectively) had parents from different ethnic backgrounds (and so likely living in a bilingual household), but Gothenburg included a significantly higher proportion of children born to non-Swedish parents (see Table 3), most likely due to the lack of availability of an interpreter in the Stockholm study. Gothenburg children had more siblings and were later in the birth order, on average, than Stockholm children. Gothenburg children were more likely to be born preterm or beyond 41 weeks’ gestation than Stockholm children (28% vs 14% respectively, see Table 4). Due to the higher degree of ethnic diversity in the Gothenburg cohort, we explored whether any of these differences were related to parental ethnicity (e.g., via cultural preferences regarding family size). Gothenburg families where at least one parent was non-Swedish had significantly fewer children (see Table 5). There was no difference in birth order or gestational age according to parental ethnicity.

**Table 3:** Ethnic background of children in Gothenburg and Stockholm cohorts

Parents’ ethnicity	Gbg (F (%))	Stk (F (%))	χ^2^	*df*	*p*
Both Swedish	42 (44.2)	123 (59.4)			
All other ethnicities	53 (55.8)	84 (40.6)	6.078	1	**0.014**

Significant p value (p < .05) is highlighted in bold

**Table 4:** Gestational period of children in Gothenburg and Stockholm cohorts

Gestational period	Gbg (F (%))	Stk (F (%))	χ^2^	*df*	*p*
Pre term < 37 weeks	12 (12.5)	12 (5.8)			
Term 37–41 weeks	69 (71.9)	178 (86.0)			
Post term > 41 weeks	15 (15.6)	17 (8.2)	8.735		**0.013**

Significant p value (p < .05) is highlighted in bold

**Table 5:** Number of siblings and birth order in Gothenburg by parental ethnicity

	Both parents Swedish Mean (SD)	All other ethnicities Mean (SD)	t	*p*
Number of siblings	1.85 (1.189)	1.42 (0.702)	− 2.136	**0.035**
Birth order	2.23 (1.143)	1.98 (0.795)	− 1.197	0.235

Significant p value (p < .05) is highlighted in bold

With reference to Tables 1 and 2, a greater proportion of Gothenburg children had feeding problems in the first 2 years, and Stockholm children had poorer language development, poorer intellectual ability (VAB scores and IQ status), and a higher proportion of children with autism (as opposed to autistic-like condition) at T1. The increased severity of symptoms in the Stockholm cohort is likely to be due to the larger sample size as well as the method of ascertainment (i.e., from cases presenting for support, rather than from whole-population screening).

### Differences Between Those with Regressive (R) and non-regressive (NR) Autism

Just over 20% (62/303) of the combined sample of children had regressive autism (see Tables 6, 7). Those with regressive autism had a younger age when they first walked (R = 13.4; NR = 14.4 months), had a more severe language impairment at T1 (R = 71.0%; NR = 41.1%) and T2 (R = 47.4%; NR = 17.4%) and more often intellectual disability (e.g., R = 66.1%; NR = 34.5% at T2), lower mean VAB scores at T1 (R = 66.81; NR = 72.24) and T2 (R = 65.07; NR = 73.35). Despite the significantly poorer intellectual functioning overall, 28 (45.9%) of those with regressive autism had an IQ of 70 or greater at T1, 5 of whom were regarded as having average intellectual functioning (IQ ≥ 85) (see Table 8). Severity of autism was higher in the regressive group, with a higher proportion of children with autism (e.g., T1 R = 79.7%, NR = 59.6%) (as opposed to autistic-like condition (T1 R = 18.6%, NR = 24.6%)). There was no difference in levels of hyperactivity between the regressive and non-regressive cases, either at T1 or T2. The R and NR groups did not differ with regard to the rate of epilepsy; at T2, 4 (7.0%) and 18 (7.6%), respectively, had developed epilepsy (Table 7). There were also no differences in the rate of maternal disease in pregnancy (R = 48.2%, NR = 41.1%). The average age at regression was 20.13 months (SD = 9.26) with 54 children (88.5%) showing regression by the age of 24 months (see Table 9).

**Table 6:** Regression and Non-regression cases: background factors

Variables	R (n = 62)^a^	NR (n = 241)^b^	Statistic	*p*
Background				
Male (F (%))	54 (87.1)	200 (83.0)	0.614^c^	0.433
Age at T1-years (M (SD))	3.5 (0.68)	3.5 (0.76)	0.216^d^	0.829
Age at T2-years (M (SD))	5.4 (0.65)	5.4 (0.69)	0.316^d^	0.752
Parents have different ethnicity (F (%))	17 (27.4)	49 (20.5)	1.376^c^	0.241
Any NDD in first degree relative? (F (%))	24 (38.7)	95 (39.4)	0.010^c^	0.919
Number siblings (M (SD))	1.30 (0.80)	1.28 (0.90)	− 0.088^d^	0.930
Birth order (M (SD))	1.77 (0.89)	1.77 (0.85)	0.031^d^	0.975
Any maternal disease in pregnancy? (F (%))^e^	27 (48.2)	90 (41.1)	0.924^c^	0.336
Gestational age (M (SD))	39.22 (2.81)	39.11 (2.10)	− 0.317^d^	0.751
Born at term (37–41 weeks)? (F (%))	46 (74.2)	201 (83.4)	2.785^c^	0.248
Caesarean section? (F (%))	16 (25.8)	60 (25.4)	0.004^c^	0.951
Neonatal care? (F (%))	9 (14.8)	50 (21.7)	1.455^c^	0.228
Age walking − months (M (SD))	13.4 (2.72)	14.4 (4.51)	2.188^d^	**0.030**
Toe walking? (F (%))	24 (38.7)	74 (30.7)	1.444^c^	0.230
Feeding problems first 2 years? (F (%))	18 (29.0)	66 (27.4)	0.067^c^	0.796

Significant p value (p < .01) is highlighted in bold

*NDD* Neurodevelopmental Disorder

^a^Specific N for each variable ranged from 56 to 62

^b^Specific N for each variable ranged from 219 to 241

^c^chi-squared test

^d^t-test

^e^Gbg data from single variable; Stk data compiled from more detailed data: chronic condition, acute condition, and/or pharmacological intervention in pregnancy

**Table 7:** Regression and Non-regression cases: measurement and assessment outcomes

Variables	R (n = 62)^a^	NR (n = 241)^b^	Statistic	*p*
Measurements				
Birthweight (M (SD))	3431.60 (662.46)	3493.85 (636.31)	0.680^d^	0.797
Birthweight SD for gestational age (F (%))			0.277^c^	0.871
≤ 2SDs	1 (1.6)	5 (2.2)		
within ± 2SDs	60 (96.8)	221 (95.3)		
>+ 2SDs	1 (1.6)	6 (2.6)		
Head Circumference (HC) Birth (M (SD))	34.88 (3.15)	34.97 (1.76)	0.235^d^	0.815
HC Birth SD for gestational age (F (%))			1.342^c^	0.511
< 2SDs	1 (1.7)	2 (0.9)		
within ± 2SDs	58 (98.3)	218 (97.3)		
≥ 2SDs	0 (0)	4 (1.8)		
HC T1 (M (SD))	51.33 (1.94)	51.19 (1.83)	− 0.498^d^	0.619
HC T1 SD (F (%))			3.613^c^	0.164
< 2SDs	1 (1.8)	2 (0.9)		
within ± 2SDs	54 (98.2)	212 (93.4)		
≥ 2SDs	0 (0)	13 (5.7)		
Assessment outcomes				
Language T1 − minimal or none (F (%))	44 (71)	99 (41.1)	17.667^c^	< **0.001**
Language T2 − minimal or none (F (%))	27 (47.4)	41 (17.4)	23.178^c^	< **0.001**
Activity level T1 − hyper/hypo (F (%))	25/3 (40.3/4.8)	98/7 (40.7/2.9)	0.583^c^	0.747
Activity level T2 − hyper/hypo (F (%))	22/3 (38.6/5.3)	117/8 (49.6/3.4)	2.736^c^	0.305
Epilepsy T1 (F (%))	4 (4.1)	16 (6.6)	0.003^c^	0.958
Epilepsy T2 (F (%))	4 (7.0)	18 (7.6)	0.020^c^	0.888
Intellectual level T1 − BIF/IDD (F (%))	23/33 (37.7/54.1)	82/75 (34.0/31.1)	19.115^c^	< **0.001**
Intellectual level T2 − BIF/IDD (F (%))	12/37 (21.4/66.1)	42/81 (17.9/34.5)	25.118^c^	< **0.001**
Vineland total T1 (M (SD))	66.81 (10.76)	72.24 (10.77)	3.420^d^	**0.001**
Vineland total T2 (M (SD))	65.07 (14.49)	73.35 (14.12)	3.939^d^	< **0.001**
ASD dx T1 − autism/autistic-like (F (%))	47/11 (79.7/18.6)	143/59 (59.6/24.6)	10.976^c^	**0.004**
ASD dx T2 autism/autistic-like (F (%))	43/12 (75.4/21.1)	110/90 (46.6/38.1)	16.039^c^	< **0.001**

Significant p values (p < .01) are highlighted in bold

*BIF* borderline intellectual functioning, *IDD* intellectual development disorder,, *ASD* autism spectrum disorder

^a^Specific N for each variable ranged from 55 to 62

^b^Specific N for each variable ranged from 224 to 241

^c^chi-squared test

^d^t-test

^e^Gbg data from single variable; Stk data compiled from more detailed data: chronic condition, acute condition, and/or pharmacological intervention in pregnancy

**Table 8:** Intellectual function in regression and non-regression cases

Intellectual level	R F (%)	NR F (%)	χ^2^	*p*
T1				
AIF	5 (8.2)	84 (34.9)		
BIF	23 (37.7)	82 (34.0)		
IDD	33 (54.1)	75 (31.1)	19.115	< **0.001**
T2				
AIF	7 (12.5)	112 (47.7)		
BIF	12 (21.4)	42 (17.9)		
IDD	37 (66.1)	81 (34.5)	25.118	< **0.001**

Note: Significant p values (p < .01) are highlighted in bold

**Table 9:** Age at regression

	Min	Max	Median	IQR	Mean	SD
Age of regression (months) n = 61	11	63	18	15–21.5	20.13	9.262

### Regression ‘From Normal’ Versus ‘From Low’

Within the Stockholm sample, where more detailed coding was applied, 20/46 children classified as regressive had followed an apparently normal developmental progression prior to regression (whereas the other 26 had regressed from an initial low level of functioning). This is partially reflected in the language production data, where significantly fewer of the ‘from normal’ group had no or minimal spoken words at T1 compared to the ‘from low’ group (50.0% vs 84.6%; *χ*^2^(1) = 6.398; *p* = .011), whereas this difference was non-significant by T2. There was no significant difference in age at first walking, level of intellectual functioning, or severity of autism between these subgroups.

### Genetic Etiology (Stockholm Only)

As Gothenburg genetic analysis is ongoing for the total sample, we will report only the Stockholm figures here. In Stockholm, 169 participants provided genetic samples and 33 (20.0%) showed clear genetic abnormalities. A further 3 were assessed clinically as having tuberous sclerosis (bringing the proportion of known genetic etiology to 20.9%). Of those with regressive autism, 40 (87%) of the 46 cases in the sample underwent genetic testing. Ten (25%) of these showed a clear genetic abnormality, including 3 with a CNV and 1 with Rett syndrome. A further 3 individuals had identified underlying etiology with a normal genetic outcome: 1 had been born extremely preterm (24 weeks), 1 had encephalitis, and 1 had neuroblastoma. Therefore, of the 46 cases of regressive autism in the Stockholm group, 13 (28.3%) had a clearly identified underlying etiology. As Table 10 shows, there was no significant difference in the proportion with a genetic abnormality in the R and NR groups within the Stockholm sample.

**Table 10:** Genetic etiology in regression and non-regression cases (Stockholm sample)

Genetic abnormality identified	R F (%)	NR F (%)	χ^2^	*df*	*p*
Yes	10 (25.0)	28 (21.2)			
No	30 (75.0)	104 (78.8)	0.613	1	0.613

There were two cases of Rett syndrome within the Stockholm sample. One had regressive autism (from normal) and the other did not. There was little difference between the two cases apart from the girl with regressive autism apparently showing greater regression within the time of measurement (i.e., from T1 to T2 her intellectual functioning moved from BIF to IDD).

## Discussion

This study aimed to describe systematically a sub-population of children with ASD and early developmental regression derived from a whole population base in Sweden’s two largest cities, and to examine how those with early regression compare to those without. We have chosen to focus on language regression specifically (rather than social, play or motor regression) as communication is by far the most common skill to be lost or diminished in regressive autism (Gadow et al. 2017). However, in children younger than 15 months, regression was determined when there was a clear indication of loss of social interest and contact. We believe that this is one of the very few relatively large-scale in-depth population studies of ‘regressive autism’. In a recent review, for example, 12 of the 85 articles meeting inclusion criteria were described as ‘population’ based (although it is impossible to tell from the review article how many of these involved whole population screening) (Barger et al. 2013). In our rare example of a large-scale population-based study of children with regressive autism, we found that regression occurs in about one in five of all children with ASD and that about half this proportion constitutes “regression from normal”. The regressive and non-regressive groups differ in expected ways—in general, poorer language development, poorer intellectual function, and greater severity of autism in those with regression. We also found that children with regressive autism began walking at an earlier age than children with non-regressive autism supporting the notion that many of them had indeed had ‘normal’ early development before the regression occurred. There were no differences between those who regressed from a relatively normal level of functioning and those who were already experiencing some impairment, with the exception of language production which was less impaired in the ‘from normal’ subgroup.

The rate of epilepsy among children in the regressive compared to the non-regressive group was similar, around 7%, a finding supported by the study from Baird et al. (2006), reporting no significant difference in epileptiform activities between children with regression and no regression. There was also a similar level of maternal disease in pregnancy in the regressive and non-regressive groups, suggesting that prenatal exposure via maternal disease does not seem to be a key feature in the development of regressive autism. Only 13 of the 46 Stockholm regressive autism cases (28%) had a clearly identified underlying etiology, a similar proportion to those without regression, with similar proportions (80/87%) undergoing genetic testing (chromosomal microarray) in either group.

The prevalence rate of around 20% in this population is in keeping with that found in previous research, e.g., Barger and colleagues (2013) found an average prevalence rate of 21.8% among population samples (as opposed to 33.6% from clinical samples). The poorer functioning, especially in relation to language skills, is also in keeping with previous literature. We have also confirmed the finding that children with regressive autism walk at an earlier age, previously shown in a smaller, clinically-derived sample (Jones and Campbell 2010).

It is important to consider issues of case ascertainment, screening and assessment approaches, and definition of regression in placing this study within the extant literature. It is a limitation of the present study that the Stockholm group was recruited on the basis of the diagnosis of ASD and the Gothenburg group after screening *and* diagnosis render conclusions about the representativity of the samples a bit difficult. Nevertheless, both groups were originally community (rather than clinic) based and relatively large, and the results are likely to be more representative of ASD than those of previously published studies that have almost always been clinic based with no clue as to the basis of recruitment in the general population. We used language regression specifically for defining regression (as opposed to language / social regression or ‘mixed’ regression—see Barger et al. 2013) but this has not been applied in exactly the same manner in the two sub-cohorts. Gothenburg cases were classified according to the timing of regression (i.e., before or after 24 months) whereas Stockholm cases were classified according to functional level (i.e., from a “normal” or “low” level, mostly close to the same time, around the age of 24 months). Application of a generally agreed definition of regression, as well as a standardised assessment protocol, would enhance our ability to generate large datasets for robust analyses.

Ozonoff et al. (2018) studied aspects concerning the definition of regression in autism. A finding was that regressive forms of onset in autism might be under-reported using certain methods. Four measures were studied; informant (examiner vs. parent), the decision type (categorical [regression absent or present] vs. dimensional [frequency of social behaviors]), and the timing of the assessment (retrospective vs. prospective). A finding in their study was that the accuracy of widely used methods of measuring onset is questionable and that the present findings argue against their widespread use.

This study nevertheless presents a rare examination of two community-based samples using similar assessment protocols both at original assessment and at follow-up, and with in-depth examinations of all cases. Future research should address the limitations around case ascertainment, method of screening / diagnosis and definition of regression, as well as expand to even larger population-based cohorts and include long term follow-up. With regard to the underlying cause/s of early regressive autism, we are in need of large collaborative studies to further explore immunological, metabolic and genetic mechanisms, synaptic and neurotransmitter signaling abnormalities, and other possible etiopathological mechanisms.

In a recent paper, Thurm et al. (2018) reported from a meeting convened by the National Institutes of Health (NIH) in 2016 on: “Loss of Skills and Onset Patterns in Neurodevelopmental Disorders: Understanding the Neurobiological Mechanisms”. With regard to recommendations and next steps, the authors discussed that regression is not a discrete event but a process, and it is dimensional rather than categorical. The need to understand the various neurobiological processes of synaptogenesis and pruning in ASD, and other neurodevelopmental disorders, using different models, including novel non-invasive methods was highlighted.

### Clinical Implications

Regression is not uncommon in ASD (occurring in about one in five of all cases of which half represent “regression from normal”), but sufficiently frequent to warrant careful consideration in all new cases with autism. Further, there is a need to make holistic and follow-up examinations of all children with ASD and perhaps particularly detailed assessments of all those in whom there is suspicion or proof of regression/loss of skills. Children with a regressive developmental trajectory, with or without autism, always need a careful neuropediatric work-up to investigate possible neurological disorders that may lead to developmental regression, taking into account possible treatable conditions.
